# Phenotypic Variation and Classification of Melon Germplasm Resources Based on DUS Test Characteristics

**DOI:** 10.3390/plants15152384

**Published:** 2026-08-03

**Authors:** Yundan Cong, Guorong Yan, Zhongqing Li, Chaohong Deng, Yue Ma, Fan Wang, Lianjia Zhao, Ning Liu, Wei Wang, Yu Song

**Affiliations:** 1 Crop Research Institute, Academy of Agricultural Sciences of Xinjiang Uyghur, Urumqi 830091, China; yundan_cong@163.com (Y.C.);; 2 New Plant Variety Testing (Urumqi) Branch Center, Ministry of Agriculture and Rural Affairs, Urumqi 830091, China

**Keywords:** muskmelon, germplasm resources, DUS test, genetic diversity, correlation analysis

## Abstract

Melon (*Cucumis melo* L.) germplasm resources are vital for seed-industry innovation and cultivar breeding. To systematically characterize their phenotypic variation, classification relationships, and breeding potential, 403 melon accessions were evaluated using nine quantitative characteristics specified in the guidelines for tests of distinctness, uniformity, and stability. These characteristics included fruit longitudinal diameter, fruit transverse diameter, fruit peduncle length, internode length, flesh thickness, petiole length, rind thickness, soluble solids content, and seed cavity diameter. The evaluated population exhibited extensive phenotypic variation, with coefficients of variation ranging from 14.0% to 57.4%. Rind thickness showed the greatest variation, while fruit transverse diameter, flesh thickness, fruit peduncle length, and fruit longitudinal diameter also displayed considerable differentiation. The Shannon–Weaver diversity index ranged from 1.74 to 1.96, indicating substantial diversity in fruit morphology, vegetative growth, internal fruit structure, and quality-related characteristics. Spearman rank correlation analysis revealed close developmental relationships among fruit-related characteristics, with rind thickness showing the strongest positive correlation with flesh thickness. Principal component analysis extracted three components with eigenvalues greater than one, which together explained 65.22% of the total phenotypic variation and primarily represented fruit morphological structure, vegetative growth, and fruit quality. Hierarchical cluster analysis classified the 403 accessions into four quantitative phenotypic groups, with highly significant differences among the groups for all nine characteristics. Furthermore, a weighted comprehensive selection index identified several accessions with superior combined agronomic performance, among which accession 20225110454A ranked first. These findings provide a quantitative phenotypic basis for melon germplasm conservation, core collection development, parental selection, and breeding-oriented resource utilization.

## 1. Introduction

Melon (*Cucumis melo* L.) is an important horticultural crop in the Cucurbitaceae family and is widely cultivated worldwide owing to its rich phenotypic diversity and high economic value [1,2]. China is currently the world’s largest melon producer, supported by a long history of cultivation and by archaeobotanical evidence from late Neolithic sites dating back to 3000–2000 BC [3]. Over centuries, germplasm introductions along varied geographic routes, combined with continuous local selection, led to the development of diverse oriental thin-skinned and western thick-skinned types [4]. According to 2023 FAOSTAT data, mainland China harvested approximately 395,800 ha of melon with a total production of 13.17 million tonnes (averaging 33.3 tonnes per hectare) [5], with leading production areas concentrated in Shandong, Xinjiang, Henan, Gansu, Hebei, and Jiangsu [6]. Among these key regions, Xinjiang represents a primary production center [7]. Its abundant solar radiation, large diurnal temperature variation, and arid climate provide favorable conditions for sugar accumulation and flavor formation in melon fruits. Under long-term natural selection, artificial domestication, and local cultivation practices, Xinjiang has developed a diverse collection of melon germplasm resources [4]. These accessions exhibit remarkable variation in fruit morphology, flesh thickness, maturity, soluble solids content, and environmental adaptability, providing essential genetic materials for germplasm conservation and targeted breeding programs [8].

The protection of plant varieties constitutes a fundamental component of agricultural intellectual property rights, serving as an essential institutional framework to promote breeding innovation and safeguard breeders’ interests [9,10,11]. The DUS (Distinctness, Uniformity, and Stability) test is a prerequisite for granting these rights [12]. It involves a standardized evaluation of morphological, biological, and agronomic traits to determine whether a candidate variety is clearly distinguishable from existing reference varieties, while maintaining uniformity within a population and stability across generations [13,14,15].

Although phenotypic DUS testing remains the cornerstone for plant variety protection [12,16,17], recent evaluations suggest that relying solely on phenotypic DUS can be insufficient for comprehensive variety characterization, highlighting the need for integrative approaches [18]. Nevertheless, owing to its standardized methodology, operational feasibility, and high repeatability [19,20], DUS testing continues to provide an indispensable morphological baseline for authenticity identification and germplasm evaluation [21,22,23].

DUS characteristics are generally classified into qualitative, pseudo-qualitative, and quantitative traits [24]. Among them, quantitative traits, such as fruit length, fruit diameter, fruit weight, flesh thickness, and soluble solids content, display continuous variation within a defined range. These traits are usually controlled by multiple genes and are sensitive to environmental conditions, thereby providing an integrated reflection of phenotypic divergence and genetic variation among germplasm resources [25]. In DUS testing, quantitative traits are commonly recorded through direct measurement, counting, or weighing, and are then described according to standardized grading criteria [24,26]. Therefore, phenotypic variation analysis based on DUS quantitative traits not only helps clarify differences among germplasm resources but also provides important references for germplasm classification, core collection construction, and parental selection in breeding programs.

However, the widespread adoption of modern commercial cultivars and intensified agricultural practices have placed indigenous melon landraces under considerable pressure [27]. The risk of germplasm homogenization and the loss of distinctive local resources is gradually increasing. Systematically evaluating the phenotypic traits of Xinjiang melon germplasm is therefore essential to identify elite accessions, conserve unique local landraces, and broaden the genetic base for future breeding efforts. Compared to molecular marker and genomic analyses, phenotypic evaluation remains intuitive, cost-effective, and highly applicable to practical breeding, making it particularly suitable for initial resource screening and variety classification.

Current research on melon germplasm primarily focuses on quality evaluation, cultivation techniques, disease resistance, and molecular diversity [28,29]. However, there remains a notable lack of systematic phenotypic variation and taxonomic studies based strictly on DUS descriptors, particularly quantitative traits. Existing evaluations often focus on a limited number of traits or localized populations, lacking comprehensive analyses of frequency distributions, diversity indices, trait correlations, principal component structures, and cluster classifications across diverse melon accessions. This gap restricts the broader application of DUS traits in the precise evaluation and breeding utilization of melon germplasm.

**Figure 1:**
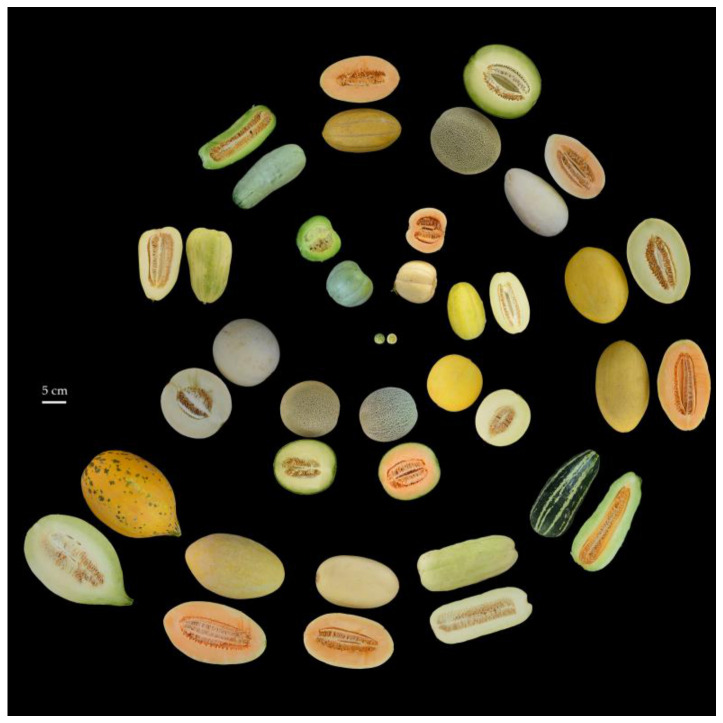
Representative fruit morphological diversity of Xinjiang melon (*Cucumis melo* L.) accessions. Note: Representative accessions are shown using paired images of the external fruit appearance and fruit section. The accessions exhibit extensive variation in fruit size and shape, including globular, ovoid, and elongated types, as well as differences in rind ground color, surface netting, stripe pattern, flesh color, flesh thickness, and seed cavity size. Some small-fruited accessions belong to the medicinal melon type locally known as “Mapao melon”; their small fruit size is an inherent phenotypic characteristic. Scale bar = 5 cm.

To address this, the present study utilizes melon germplasm resources from Xinjiang as the research subject (Figure 1). Based on quantitative trait data derived from standard DUS testing, we conducted comprehensive statistical evaluations—including frequency distribution, genetic diversity, correlation, principal component, and cluster analyses—to systematically characterize phenotypic variation and taxonomic relationships. The objective of this study is to elucidate the variation patterns of quantitative traits in Xinjiang melon germplasm, analyze the correlations among key agronomic traits, reveal phenotypic differentiation and population structure, and identify elite accessions with high breeding potential. These findings aim to provide a theoretical foundation and material basis for the conservation, core collection construction, parental selection, and targeted breeding of melon resources in Xinjiang.

## 2. Results

### 2.1. Variation Characteristics of Quantitative DUS Traits in Melon Germplasm Resources

**Figure 2:**
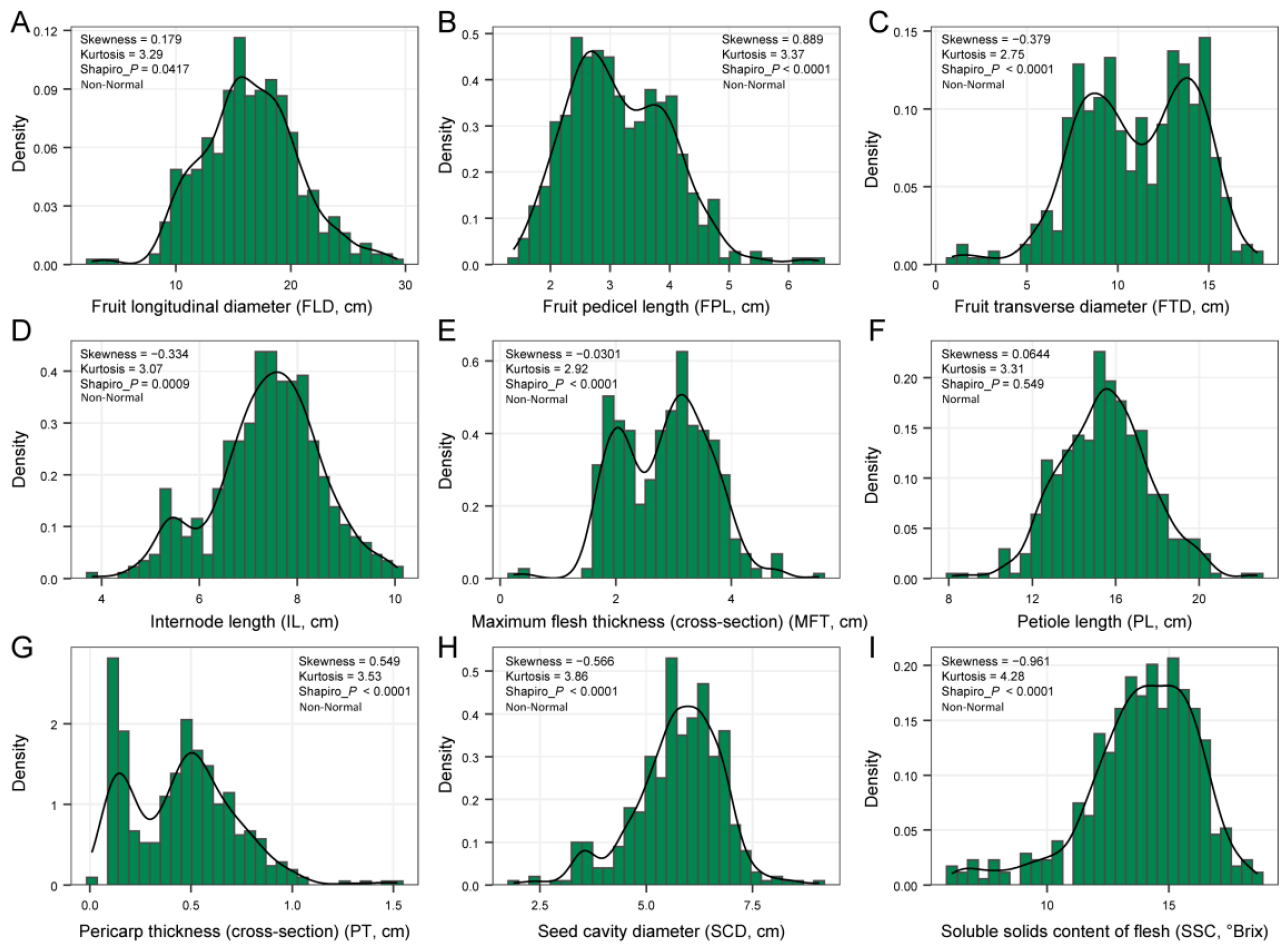
Probability density distributions of nine quantitative DUS traits in 403 melon germplasm accessions. Note: Histograms show the density distributions of (**A**) fruit longitudinal diameter (FLD), (**B**) fruit pedicel length (FPL), (**C**) fruit transverse diameter (FTD), (**D**) internode length (IL), (**E**) maximum flesh thickness in cross-section (MFT), (**F**) petiole length (PL), (**G**) pericarp thickness in cross-section (PT), (**H**) seed cavity diameter (SCD), and (**I**) soluble solids content of flesh (SSC). Black curves represent kernel density estimates. The Shapiro–Wilk test was used to assess normality. Traits with *p* ≥ 0.05 were considered approximately normally distributed, whereas traits with *p* < 0.05 were considered non-normal.

To clarify the phenotypic variation characteristics of quantitative traits in DUS melon germplasm resources, we conducted descriptive statistical analysis on 9 quantitative traits of 403 melon resources, including fruit longitudinal diameter (FLD), fruit pedicel length (FPL), fruit transverse diameter (FTD), internode length (IL), flesh thickness (MFT), petiole length (PL), rind thickness (PT), soluble solid content (SSC), and seed cavity diameter (SCD). The results indicated abundant phenotypic variation across all traits, with coefficients of variation (CV) ranging from 14.0% to 57.4% (Supplementary Table S1). Specifically, PT showed the most pronounced differentiation, ranging from 0.01 to 1.52 cm and exhibiting the highest CV of 57.4%. In addition, FTD, MFT, FPL, and FLD also showed relatively high variation, with CVs greater than 25.0%, whereas PL and IL were comparatively stable, with CVs of 14.0% and 14.7%, respectively. Regarding distribution profiles, the traits displayed distinct skewness and kurtosis patterns (Figure 2), with SSC showing a marked left-skewed distribution (skewness = −30.961, kurtosis = 4.28, Figure 2I), indicating a high density of high-sugar accessions. The Shapiro–Wilk test further confirmed that, except for PL (*p* = 0.549, Figure 2F), which strictly conformed to a normal distribution, the remaining eight traits significantly deviated from normality (*p* < 0.05).

### 2.2. Phenotypic Diversity Analysis of Quantitative DUS Characteristics

**Figure 3:**
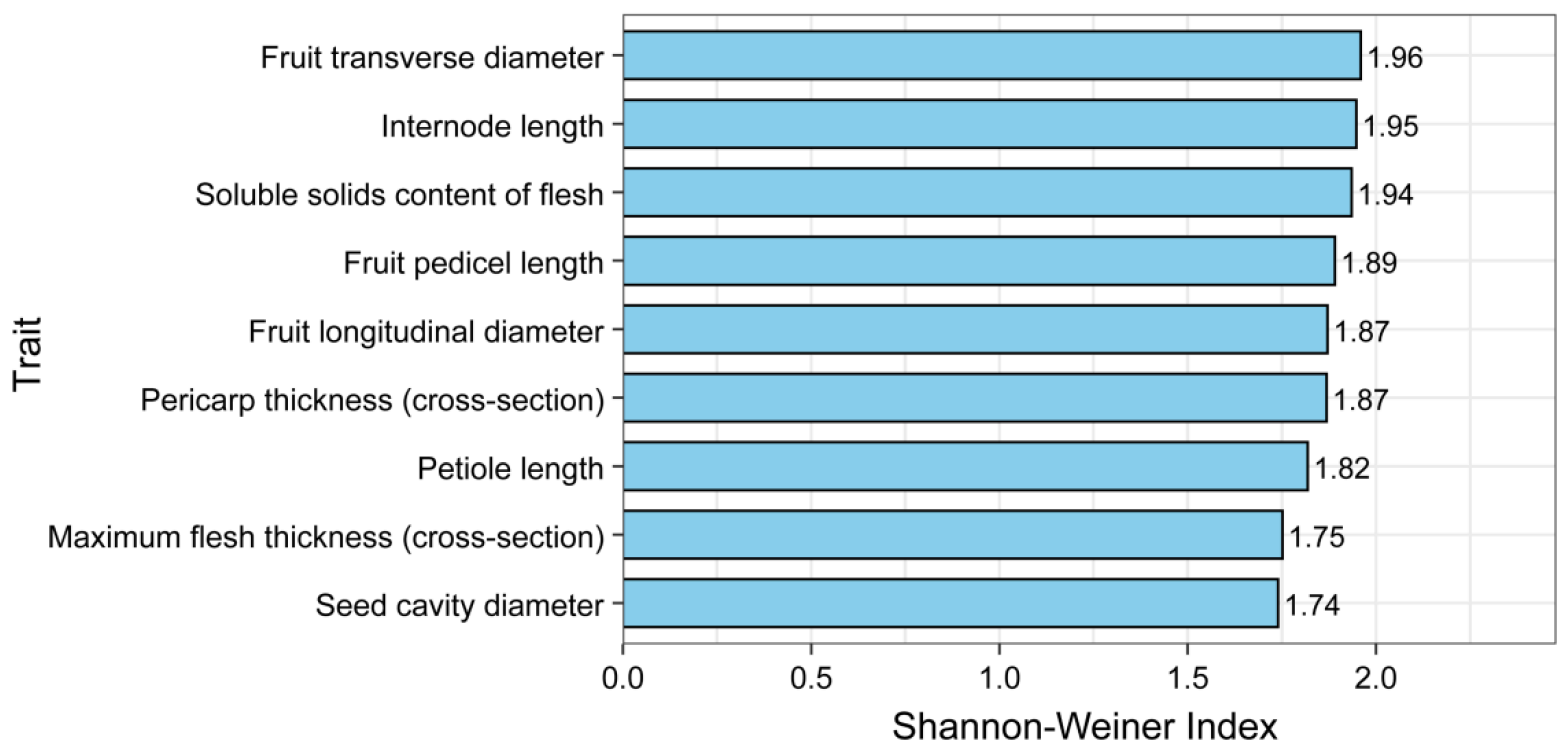
Shannon–Weaver Index of 9 quantitative traits in 403 melon germplasm.

To further clarify the diversity level of 403 melon germplasm resources in DUS quantitative traits, we conducted Shannon–Weaver diversity index analysis on 9 quantitative traits. The results revealed a generally high level of phenotypic diversity, with indices ranging from 1.74 to 1.96 (Figure 3), confirming abundant variation within the tested population. Specifically, FTD exhibited the highest diversity index (1.96), followed closely by IL (1.95) and SSC (1.94), suggesting that these traits possess rich phenotypic types and balanced distributions, making them critical indicators for germplasm discrimination. High diversity was also maintained in FPL (1.89), FLD (1.87), and PT (1.87). In contrast, relatively lower diversity indices were recorded for SCD (1.74) and MFT (1.75), indicating weaker population differentiation. In conclusion, the tested melon population possesses high phenotypic diversity in fruit morphology, quality, and internal structures, which serve as crucial indicators for germplasm discrimination, whereas vegetative growth traits are more conserved; these findings provide a valuable phenotypic foundation for melon classification, elite germplasm screening, and novel cultivar breeding.

### 2.3. Correlation Patterns Among Quantitative Traits in Melon Germplasm

**Figure 4:**
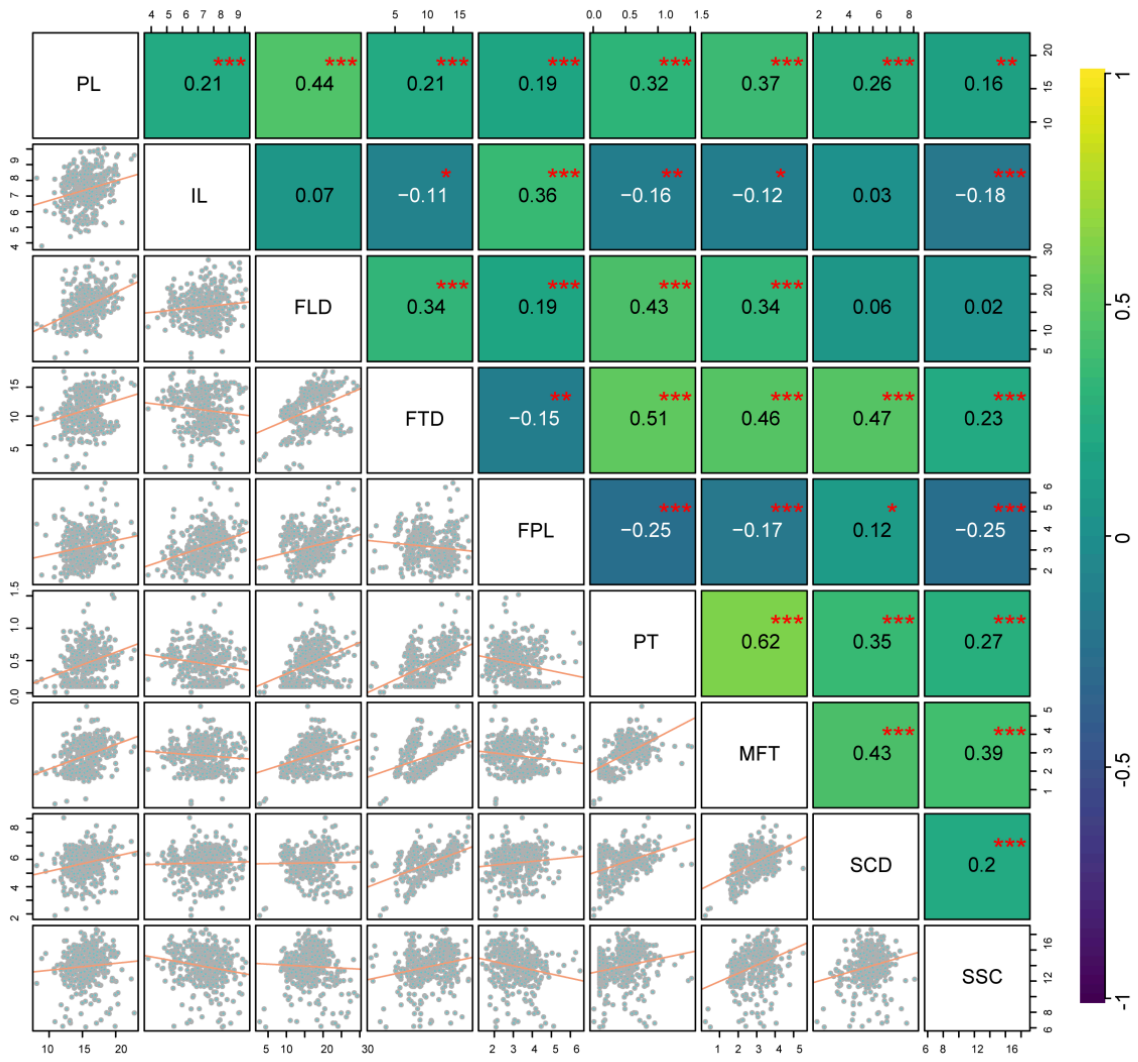
Correlation analysis of 9 quantitative traits in 403 melon germplasm. Note: The upper triangle shows Spearman’s rank correlation coefficients, with cell colors indicating the direction and magnitude of correlations from −1 to 1. *, **, and *** indicate significant correlations at the 0.05, 0.01, and 0.001 levels, respectively. The lower triangle presents pairwise scatterplots with fitted linear regression lines (orange lines), while trait abbreviations are displayed along the diagonal.

To clarify the interrelationships among the DUS quantitative traits, a Spearman rank correlation analysis was performed on nine quantitative traits across 403 melon germplasm accessions. The results showed varying degrees of correlation among the traits, with the closest associations observed among fruit-related traits (Figure 4). PT was most strongly and positively correlated with MFT (r = 0.62, *p* < 0.05). FTD exhibited highly significant positive correlations with PT (r = 0.51, *p* < 0.05), SCD (r = 0.47, *p* < 0.05), and MFT (r = 0.46, *p* < 0.05), indicating that lateral fruit expansion is closely linked to internal fruit structures. Additionally, MFT was positively correlated with SCD (r = 0.43, *p* < 0.05) and SSC (r = 0.39, *p* < 0.05), while PL correlated positively with FLD, MFT, and PT, suggesting a synergistic relationship between vegetative growth and fruit development. Conversely, IL was negatively correlated with SSC, PT, and FTD; similarly, FPL displayed negative correlations with SSC, PT, MFT, and FTD, pointing to potential trade-offs between certain vegetative or peduncle traits and fruit morphology/quality traits.

### 2.4. Principal Component Analysis and Major Phenotypic Factors of Melon Germplasm

**Figure 5:**
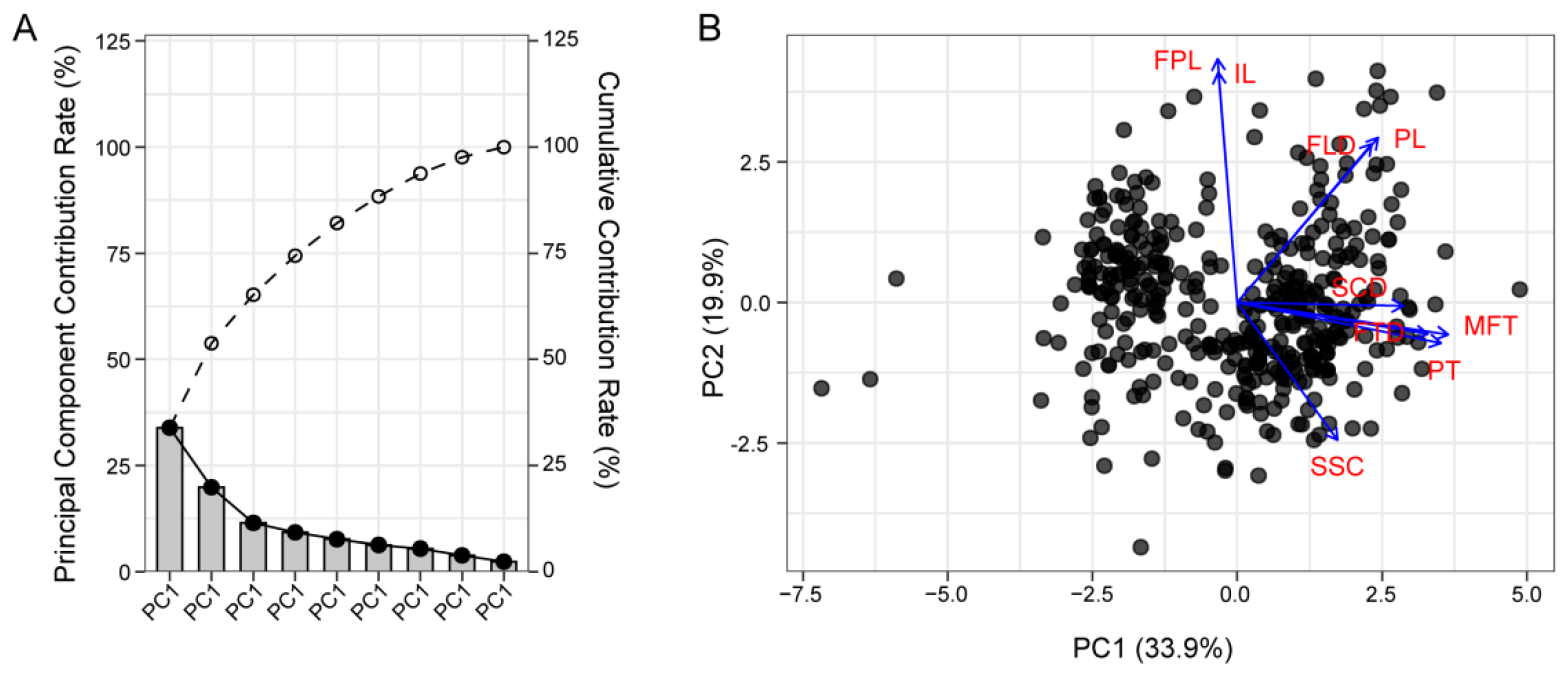
Principal component analysis of DUS test characteristics for melon varieties. Note: (**A**) Scree plot of principal components. Gray bars indicate the contribution rate of each principal component, and the dashed line with open circles represents the cumulative contribution rate. (**B**) PCA biplot of nine DUS quantitative traits across 403 melon germplasm accessions. Black points represent individual melon accessions. Blue arrows indicate the loading vectors of the evaluated traits, with arrow direction and length reflecting their associations with the principal components and their relative contributions to variation. Red labels denote trait abbreviations.

To further extract the main variant information in the quantitative traits of melon DUS, this study conducted PCA analysis on 9 quantitative traits from 403 melon germplasm resources. The results showed that the eigenvalues of the first three principal components were all greater than 1, at 3.052, 1.787, and 1.031 (Supplementary Table S2), and a cumulative contribution rate of 65.22% (Figure 5A), indicating that the first three principal components can summarize most phenotypic variation information in the test resources. Among them, PC1 is mainly contributed by traits such as MFT, PT, FTD, SCD, PL, and FLD, reflecting comprehensive variation in fruit size, peel thickness, pulp thickness, and fruit structure, which can be summarized as fruit morphology and structural factors. PC2 is mainly contributed by FPL, IL, PL, and FLD, while SSC shows a significant negative loading, indicating that this principal component mainly reflects plant growth, fruit stalk characteristics, and sugar content changes, which can be summarized as vegetative growth and quality differentiation factors; PC3 further explained some phenotypic differences not covered by PC1 and PC2 (Figure 5B). Currently, single-trait evaluations cannot fully reflect the overall performance of melon resources, whereas PCA can reduce multiple correlated traits to a few comprehensive factors, improving the overall and interpretable nature of resource evaluation. Overall, PCA results indicate that the main phenotypic differences in melon resources are concentrated in fruit morphological structure, vegetative growth, and quality traits, providing a basis for subsequent comprehensive evaluation of agronomic traits, resource classification, and screening of superior parents.

### 2.5. Cluster Analysis and Classification of Melon Germplasm Resources

**Figure 6:**
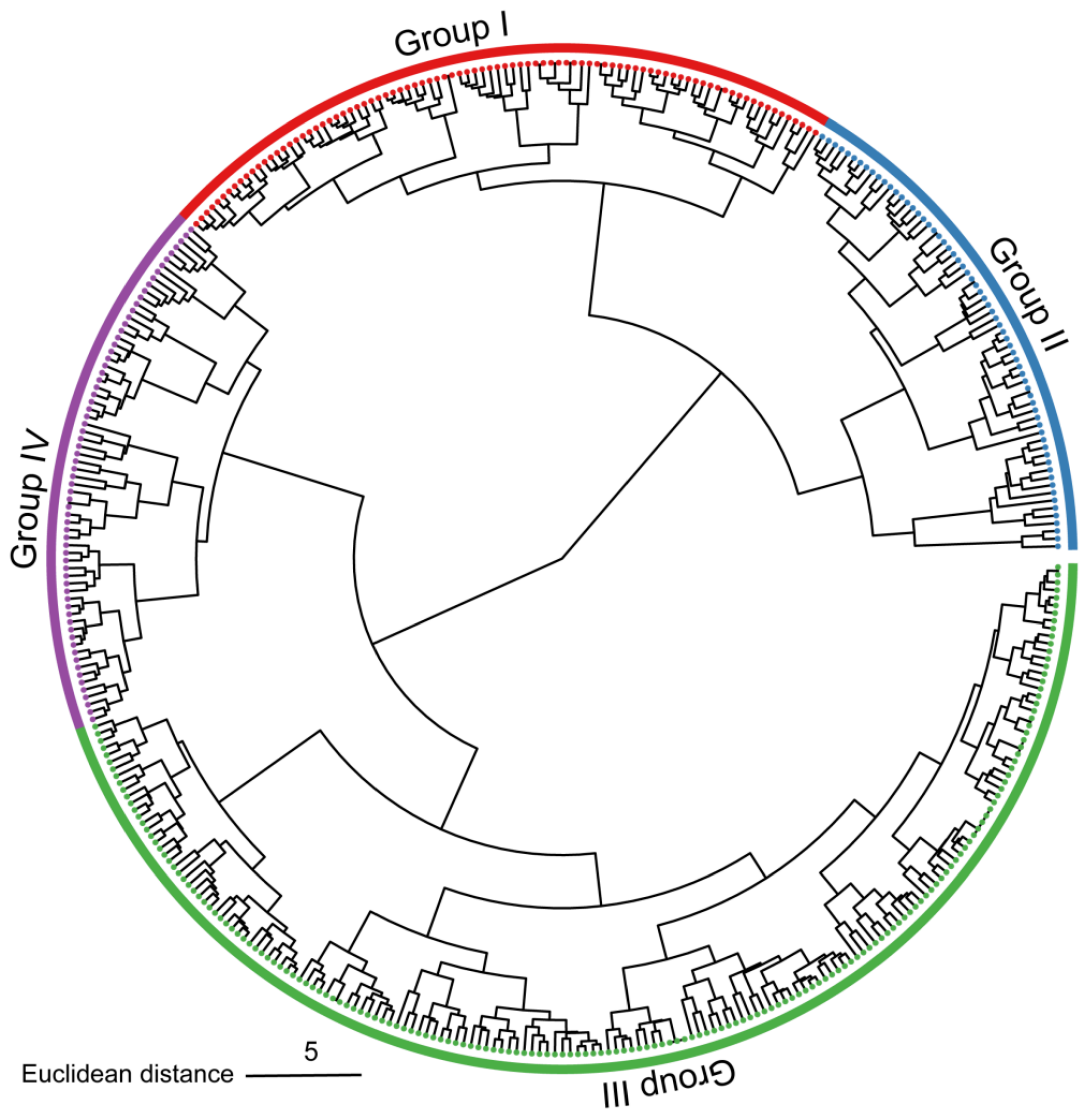
Cluster analysis of 403 melon germplasm accessions based on 9 key phenotypic characteristics. Note: The clustering was performed using Euclidean distance and the Ward.D2 method. The dendrogram was cut at a height of 19.46, resulting in four phenotypic clusters. Branch lengths represent Euclidean distances based on standardized DUS quantitative traits, and the scale bar indicates phenotypic distance units.

**Figure 7:**
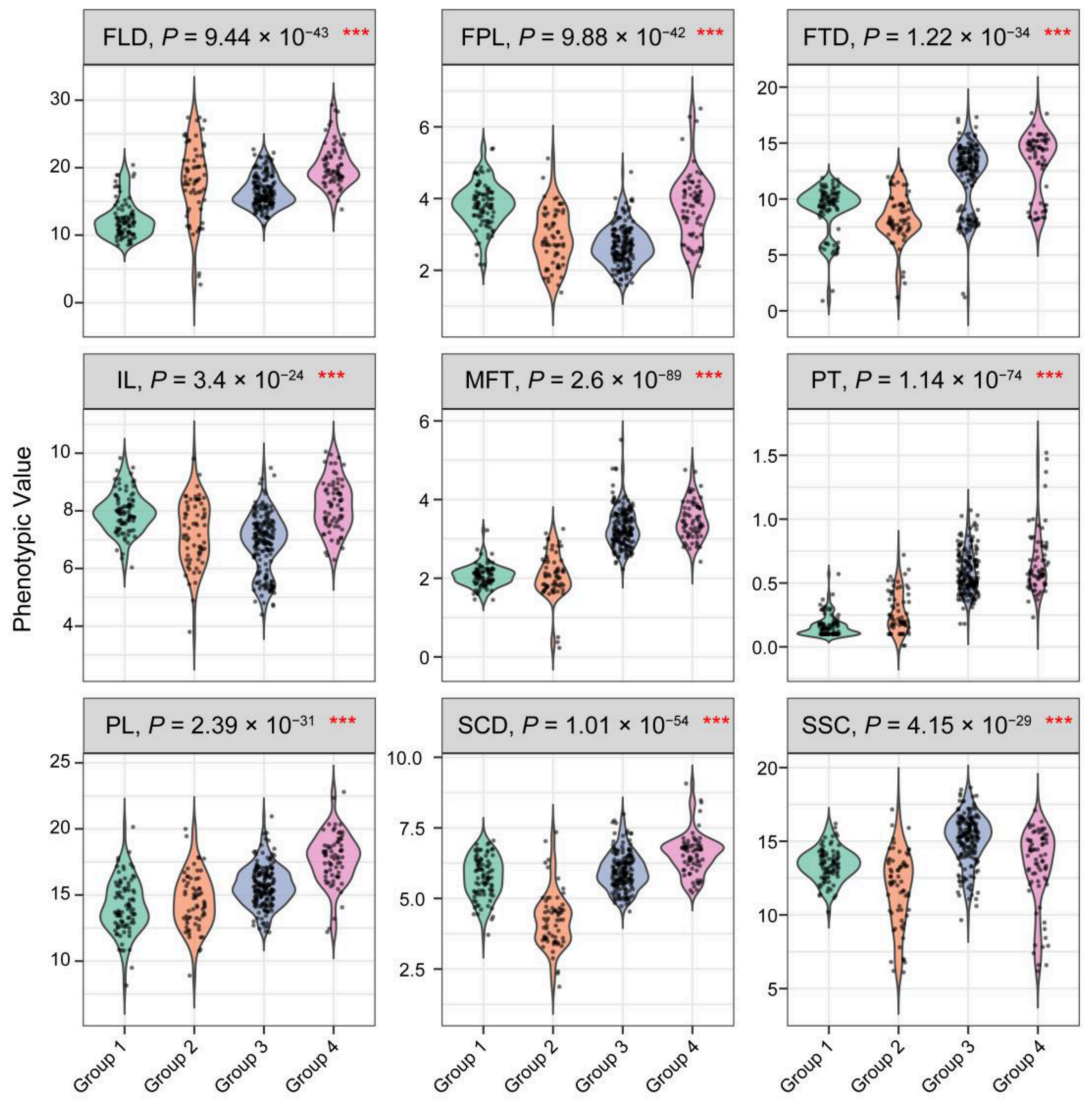
Distribution differences in quantitative DUS characteristics among different melon germplasm clusters. Note: Different colors represent four phenotypic clusters classified based on nine quantitative DUS characteristics. Violin plots show the density distribution of each quantitative DUS characteristic among clusters, and points represent phenotypic values of individual germplasm accessions. ANOVA results are shown in each panel. *** indicates highly significant differences among clusters at *p* < 0.001.

To further clarify the phenotypic classification relationships of 403 melon germplasm resources, this study conducted a systematic cluster analysis based on nine standardized DUS quantitative trait data using Euclidean distance. To determine an appropriate clustering scheme, the average silhouette width was calculated for different cluster numbers from k = 2 to k = 10 (Figure S1). Although k = 2 showed the highest average silhouette width, this classification only reflected broad phenotypic differentiation among the tested accessions (Supplementary Table S3). Based on the silhouette results, dendrogram structure, phenotypic interpretability, and subsequent group difference tests, k = 4 was selected as the final clustering scheme. Accordingly, the dendrogram was cut at a height of 19.46, dividing the 403 melon germplasm accessions into four phenotypic groups, with Group I containing 89 materials, Group II containing 65 materials, Group III containing 180 materials, and Group IV containing 69 materials, indicating significant cluster differentiation among different melon resources in DUS quantitative traits (Figure 6). One-way ANOVA further revealed that all nine traits exhibited extremely significant differences among the four clusters (Figure 7, *p* < 0.001), confirming a highly robust phenotypic discrimination. Specifically, MFT demonstrated the most pronounced variation among groups (F = 241.0, *p* = 2.60 × 10^−89^), followed by PT (F = 183.0, *p* = 1.14 × 10^−74^) and SCD (F = 118.0, *p* = 1.01 × 10^−54^), identifying them as the primary traits driving group differentiation. Highly significant differences were also observed for FLD, FPL, FTD, IL, PL, and SSC, indicating that fruit architecture, peduncle characteristics, vegetative growth, and quality traits collectively shape the phenotypic divergence of the melon germplasm. Accordingly, the 403 melon germplasm accessions were divided into four quantitative phenotypic groups under the tested environmental conditions. These groups reflect overall similarity in the measured DUS quantitative traits and should not be interpreted as direct evidence of inherent genetic classification.

### 2.6. Comprehensive Evaluation of Agronomic Characteristics in DUS Testing

**Figure 8:**
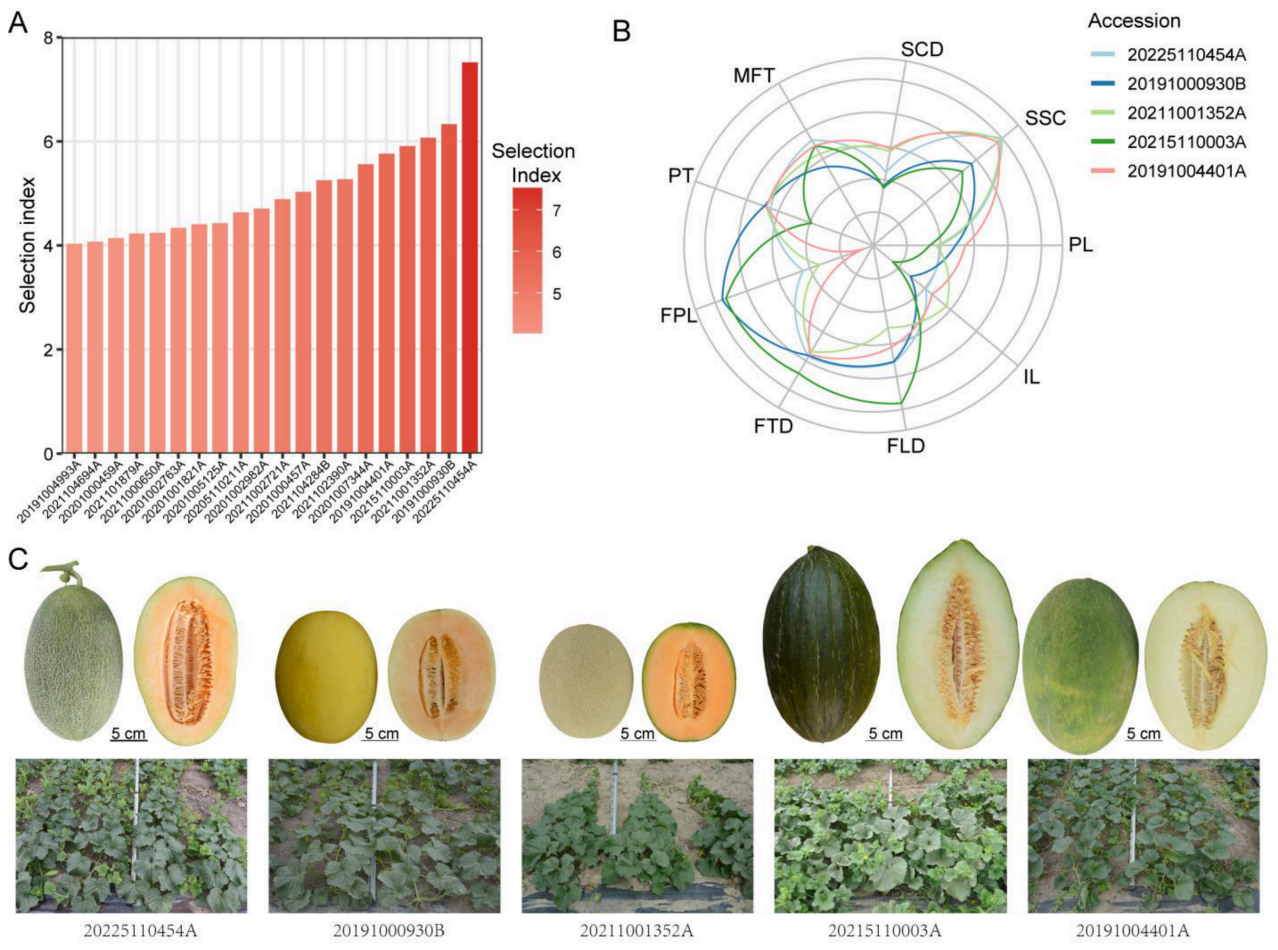
Multi-trait selection identifies elite melon accessions with complementary phenotypic profiles. Note: (**A**) Ranking of the 20 highest-scoring melon accessions according to the composite selection index. Accessions are arranged in ascending order of selection index, with darker bar colors indicating higher index values. (**B**) Radar plots showing the normalized values of nine quantitative DUS traits for the five top-ranked accessions: 20225110454A, 20191000930B, 20211001352A, 20215110003A, and 20191004401A. (**C**) Representative whole-fruit morphology, longitudinal fruit sections, and field growth performance of the five selected accessions, presented from left to right in the same order as in panel (**B**). Scale bars in the fruit images represent 5 cm.

To identify melon germplasm accessions with superior comprehensive agronomic performance, a weighted comprehensive selection index (SI) was constructed based on specific breeding targets. To select for a compact plant architecture, large fruit size, thick flesh, high sugar content, thin pericarp, and small seed cavities, FLD, FTD, FPL, MFT, and SSC were designated as positive selection traits, whereas PL, IL, PT, and SCD were designated as negative selection traits, with weights assigned as follows: SSC = 2; FLD, FTD, FPL, PT, MFT, and SCD = 1; PL and IL = 0.5. The results revealed differentiation in SI across the 403 accessions (Supplementary Table S4), leading to the successful identification of the top five elite materials: 20225110454A (7.518), 20191000930B (6.330), 20211001352A (6.070), 20215110003A (5.909), and 20191004401A (5.763). Notably, the top-ranked accession exhibited prominent phenotypic advantages, simultaneously combining high SSC, large fruit size (FLD and FTD), and thick MFT (Figure 8). However, a high index value should not be interpreted as direct confirmation of general breeding superiority. The candidate accessions require further evaluation through multi-environment trials, additional quality and resistance measurements, combining-ability analysis, and progeny or hybrid-performance testing. In conclusion, this index effectively overcomes the limitations of single-trait selection, and the screened high-scoring lines represent valuable Xinjiang melon resources that can serve as pivotal materials for core collection construction, parental mating, and novel cultivar breeding.

## 3. Discussion

### 3.1. DUS Quantitative Traits Can Effectively Reveal the Phenotypic Diversity of Melon Germplasm Resources

DUS test traits are characterized by standardization, repeatability, and ease of comparison. While phenotypic DUS traits alone may have limitations in resolving closely related genotypes [14], they remain essential phenotypic criteria for preliminary germplasm resource evaluation and diversity screening [12,13]. Previous studies have shown that melons exhibit extremely rich phenotypic differentiation, particularly in fruit shape, fruit size, rind color, flesh color, and seed traits, displaying wide variations. These traits can be used to classify different horticultural groups and subgroups [30,31]. This study evaluates 403 melon germplasm resources based on DUS quantitative traits, revealing significant differences in fruit morphology, fruit structure, vegetative growth, and quality traits among the tested materials, indicating that Xinjiang melon resources possess high phenotypic diversity and utilization potential. This aligns with previous conclusions about the rich phenotypic diversity of melon resources [32,33].

Xinjiang melons have long been influenced by unique ecological environments, cultivation practices, and artificial selection, leading to significant differentiation in traits such as fruit size, flesh thickness, sugar content, rind thickness, and seed cavity size among different resources [4,8]. These differences not only reflect phenotypic characteristics between resources but may also represent adaptive outcomes of long-term domestication and local selection [34]. Therefore, phenotypic evaluation based on DUS quantitative traits is a crucial foundation for the conservation, classification, and breeding utilization of melon germplasm resources.

Previous melon germplasm studies have commonly reported extensive variation in fruit morphology and quality and have used principal component or cluster analysis to classify accessions. The present study does not contradict these findings. Its contribution lies in evaluating 403 accessions through a unified national DUS protocol over three consecutive years and integrating descriptive variation, trait correlations, principal components, phenotypic clustering, group-difference analysis, and breeding-oriented selection within the same analytical framework. This approach provides a directly comparable quantitative dataset for a large germplasm collection managed by a variety testing institution. Nevertheless, molecular data and accessions from additional geographic regions will be required to determine whether the phenotypic patterns identified here represent regional adaptation, shared horticultural types, or broader melon diversity.

### 3.2. Multivariate Comprehensive Analysis Helps Clarify the Classification of Melon Resources and the Direction of Breeding Utilization

Melon germplasm resources are numerous and diverse, making it difficult to accurately evaluate their comprehensive utility value based on a single trait. Multivariate statistical methods can integrate multiple traits for analysis, revealing overall differences among resources. Previous studies have widely used PCA and cluster analysis in evaluating melon germplasm resources to interpret sources of phenotypic variation and classify materials [34,35,36]. In Chinese melon resource research, Luan et al. [3] used diversity analysis to find that Chinese melon resources showed clear differentiation from some foreign reference resources in clustering, indicating that the classification structure of melon resources is related to geographic origin, horticultural types, and genetic background. Other studies have also shown that cluster analysis based on morphological traits or molecular markers can divide melon resources into different groups, providing a basis for germplasm resource conservation and parental selection.

In this study, correlation analysis, PCA, and cluster analysis revealed the comprehensive differences in melon resources from different angles. Correlation analysis can reflect the synergistic or trade-off relationships between fruit structure, quality, and plant growth traits; PCA can extract major comprehensive factors to clarify the main sources of resource differentiation; and cluster analysis can classify resources based on overall phenotypic similarity. Combining existing research shows that fruit-related traits such as flesh thickness, fruit size, rind thickness, seed cavity size, and soluble solid content are the most explanatory indicators in the classification and breeding utilization of melon resources. For melon breeding, materials that perform outstandingly in different groups can be used for specific trait improvement, while materials with significant differences between groups can be used to broaden the genetic basis of parental lines.

### 3.3. Comprehensive Selection Index Improves Resource Screening Efficiency

In practical breeding, excellent melon materials are not typically optimal for a single trait but rather achieve a relative coordination among multiple target traits. Previous research has also shown that the evaluation of melon resources often requires considering fruit morphology, quality, yield-related traits, and adaptive characteristics simultaneously, as single-trait selection alone cannot fully reflect the breeding value of the materials [37,38].

Our analysis sets trait directions and weights based on melon breeding objectives, constructs a comprehensive selection index, and screens candidate materials with superior overall performance. This method can integrate multiple DUS quantitative traits into a single evaluation index, helping to improve the efficiency of selecting excellent resources and reducing the bias introduced by single-trait selection. However, the results of the comprehensive selection index are closely related to breeding objectives and weight settings. The selection direction for different types of melons is not entirely consistent; fresh-consumption melons should focus more on soluble solid content, flesh thickness, and sensory-related traits, while storage-and-transport-resistant melons need to balance rind thickness, fruit structural stability, and marketability.

The comprehensive selection index provides a transparent method for integrating multiple quantitative traits, but its output represents candidate prioritization rather than final confirmation of breeding value. The present study did not include independent progeny testing or F1 hybrid evaluation. Therefore, the identified accessions should first be used as candidates for further characterization. Their breeding value should be validated by crossing them with genetically and phenotypically contrasting parents, evaluating general and specific combining ability, and testing the yield, quality, uniformity, and stability of their progenies across multiple environments. Similarly, construction of a core collection should incorporate information on geographic origin, horticultural type, qualitative traits, molecular diversity, and redundancy among accessions rather than relying solely on the present quantitative clusters.

Meanwhile, most DUS quantitative traits are complex and easily influenced by environmental conditions. Therefore, although phenotypic evaluation can directly serve breeding selection, subsequent stability verification should still be conducted through multi-year and multi-location trials. Existing research has shown that the phenotypic classification of melon resources does not always align perfectly with molecular classification. Combining morphological data with molecular markers or genomic data can help more accurately interpret the genetic relationships and classification structures of germplasm resources [3]. Therefore, future research will establish an integrated evaluation framework for Xinjiang melon germplasm by combining molecular profiling, multi-environment testing, and target-driven selection. First, high-quality genome-wide SNPs will be used to construct a targeted genotyping panel for germplasm fingerprinting, diversity assessment, and molecular-assisted selection. Second, multi-year and multi-location trials across key Xinjiang ecological regions will evaluate genotype-by-environment (*G × E*) interactions via mixed linear models to identify phenotypically stable accessions. Third, breeding-objective-tailored selection indices will be established with trait weightings customized for fresh fruit quality, storability, and early facility cultivation. Co-deploying molecular marker platforms, multi-environment stability analysis, and objective-specific selection will effectively distinguish genetic variation from environmental noise, enhancing the efficiency of parental selection and cultivar development.

### 3.4. Limitations

Although this study systematically evaluated the phenotypic variation and classification of melon germplasm resources using quantitative DUS traits, several limitations should be acknowledged.

First, all field experiments were conducted at a single experimental location. Although the accessions were evaluated over three consecutive years, spatial environmental heterogeneity across different ecological regions was not assessed. Quantitative traits may be jointly influenced by genotype, environment, and genotype-by-environment interactions, and the relative performance of germplasm accessions may change across environments [39,40]. Therefore, the four groups identified in this study should be interpreted primarily as quantitative phenotypic clusters under the tested environmental conditions rather than as stable classifications across broad ecological regions. Future studies should conduct multi-year and multi-location trials to evaluate trait stability, environmental adaptability, and the consistency of cluster membership across environments.

Second, the present analysis was based mainly on phenotypic data and lacked independent validation using molecular markers. Phenotypic divergence should not be interpreted directly as genome-wide genetic divergence because observed phenotypes may also be affected by environmental variation and genotype-by-environment interactions [41]. Future studies should integrate SNP genotyping, genome-wide resequencing, or other genomic information with standardized phenotypic observations. The joint use of genomic and phenomic data could provide a more reliable basis for evaluating genetic relationships, identifying representative germplasm, constructing core collections, and selecting parental materials [42].

Third, the comprehensive selection index was constructed using manually assigned trait directions and weights. Although this weighting scheme reflected the breeding objectives defined in the present study, the relative importance assigned to individual traits may influence accession ranking and may not be equally applicable to different horticultural types, production systems, or market demands. Multi-trait selection indices should therefore be refined according to clearly defined breeding objectives, trait variance-covariance relationships, correlated selection responses, and the desired direction of genetic improvement [43]. Differentiated indices could be developed for fresh-consumption quality, storage and transportation, early maturity, protected cultivation, and other specific breeding targets.

Fourth, only quantitative DUS traits were included in the present analysis, whereas qualitative and pseudo-qualitative characteristics were not incorporated. Traits such as fruit shape, rind color, flesh color, surface pattern, and netting are important for describing horticultural types and identifying morphologically distinct germplasm. Consequently, the four groups identified in this study represent quantitative phenotypic clusters rather than a complete horticultural classification. Future studies should integrate qualitative, pseudo-qualitative, and quantitative descriptors using analytical approaches suitable for mixed data types. Gower distance can accommodate variables measured on different scales, while multiple correspondence analysis can summarize relationships among categorical characteristics [44]. Combined analysis of qualitative and quantitative data may improve germplasm classification and the construction of representative core collections [45,46].

Overall, the integration of multi-environment phenotypic evaluation, genomic information, qualitative and quantitative descriptors, and breeding-objective-specific selection indices will provide a more comprehensive framework for melon germplasm classification, core collection construction, parental selection, and breeding utilization [42,43,45,46].

## 4. Materials and Methods

### 4.1. Plant Materials and Field Experiment

A total of 403 melon germplasm accessions were evaluated in this study. This collection, comprising 361 breeding lines and 42 commercial cultivars, was provided by the Urumqi Branch Center for Plant Variety Testing, Ministry of Agriculture and Rural Affairs, China. Field experiments were conducted from 2021 to 2023 at the Anningqu Experimental Station of the Xinjiang Academy of Agricultural Sciences (45°56′ N, 87°56′ E; altitude 590 m) in the Xinjiang Uygur Autonomous Region. The experimental site is characterized by a temperate semi-arid continental climate, with soil predominantly classified as irrigated grey desert soil of moderate fertility.

The trials were arranged in a randomized block design, with two plots serving as biological replicates for each accession. Seeds were sown uniformly between May 15 and May 18 each year. A plastic-film mulching system was employed, utilizing a film width of 0.4 m and an inter-film spacing of 3.0 m. Two drip irrigation lines were installed beneath each plastic film. Seedlings emerged approximately one-week post-sowing, and residual seed coats on the cotyledons were manually removed as needed. Thinning was performed roughly two weeks after sowing, retaining two plants per hill to maintain a minimum of 35 plants per plot. Guard rows were established around the experimental perimeter, and routine agronomic management—including irrigation, fertilization, and pest control—conformed to local conventional practices for melon production.

### 4.2. DUS Quantitative Trait Investigation

All traits were measured according to the Chinese national standard Guidelines for the Conduct of Tests for Distinctness, Uniformity and Stability: Melon (GB/T 19557.21-2022). Healthy plants or representative fruits were randomly selected from each replicated plot for trait measurement.

Petiole length (PL) and internode length (IL) were measured at the near fruit-setting stage. PL was measured as the distance from the base of the petiole of functional leaves at the 10th to 15th nodes on the main vine to the connection point with the main vine. IL was recorded as the average length of adjacent internodes. Fruit-related traits were measured at physiological maturity. Fruit longitudinal diameter (FLD) and fruit transverse diameter (FTD) were measured using a digital caliper, representing the maximum length between the two ends of the fruit and the maximum diameter at the equatorial plane, respectively. Fruit pedicel length (FPL) was measured as the distance from the natural abscission layer to the connection point with the vine. After transverse cutting of the fruit, pericarp thickness (PT), mesocarp flesh thickness (MFT), and seed cavity diameter (SCD) were immediately measured. PT was defined as the distance from the outer fruit surface to the tissue transition zone of the flesh, MFT as the maximum distance from the inner pericarp to the outer edge of the seed cavity, and SCD as the maximum diameter of the seed cavity in the transverse section.

Central soluble solids content (SSC) was measured using a handheld digital refractometer (PAL-1, Atago, Japan). Before measurement, the instrument was calibrated with distilled water to 0.0 °Brix at 20 °C. A flesh slice approximately 1 cm thick was collected from the equatorial region of each fruit, and the juice was extracted by pressing through double-layer gauze. Approximately 0.1 mL of clarified juice was transferred onto the detection prism using a micropipette. The SSC value was recorded after stabilization for 3 s and expressed as °Brix with an accuracy of 0.1.

For PL, IL, FLD, FTD, FPL, PT, MFT, and SCD, no fewer than 10 samples were measured for each replicate (*n* ≥ 10), whereas SSC was measured using no fewer than 5 samples for each replicate (*n* ≥ 5). The arithmetic mean of all measurements within each biological replicate was used as the final phenotypic value for each trait.

For each accession and trait, measurements from individual plants or fruits within each biological replicate were first averaged to obtain a replicate-level phenotypic value. The values from the two biological replicates were then averaged to calculate the annual phenotypic value of each accession. Finally, the annual values obtained in 2021, 2022, and 2023 were averaged to generate the three-year mean phenotypic value. The resulting three-year mean values were used for subsequent descriptive statistics, phenotypic diversity analysis, correlation analysis, principal component analysis, cluster analysis, analysis of variance among phenotypic groups, and comprehensive selection-index calculation.

### 4.3. Descriptive Statistics and Phenotypic Diversity Analysis

Descriptive statistical analysis was performed for nine quantitative DUS characteristics, including PL, IL, FLD, FTD, FPL, PT, MFT, SCD, and SSC. The minimum value, maximum value, mean, standard deviation (SD), coefficient of variation (CV), skewness, and kurtosis were calculated for each trait. The CV was used to evaluate the degree of dispersion of phenotypic variation among different melon germplasm accessions and was calculated as follows:

$$CV\;\left(\%\right)=\frac{SD}{Mean}\times 100$$

The Shapiro–Wilk test was used to examine the normality of each trait. Traits with *p* > 0.05 were considered normally distributed, whereas traits with *p* ≤ 0.05 were considered non-normally distributed.

Phenotypic diversity was evaluated using the Shannon–Weaver diversity index. For each trait, phenotypic values were divided into different grades according to their distribution characteristics, and the frequency of each grade was calculated. The Shannon–Weaver diversity index was calculated as follows:

$$H^{\prime }=-\sum_{i=1}^{n}P_{i}\ln P_{i}$$

where $P_{i}$ represents the proportion of accessions in the *i*th grade, and n represents the total number of grades. A higher $H^{\prime }$ value indicates greater phenotypic diversity and a more even distribution of trait variation among the tested germplasm resources.

### 4.4. Correlation Analysis and Principal Component Analysis

Correlation analysis was performed for nine quantitative DUS characteristics, including PL, IL, FLD, FTD, FPL, PT, MFT, SCD, and SSC. Since most traits did not conform to a normal distribution, Spearman’s rank correlation coefficient was used to evaluate the relationships among traits. The Spearman correlation coefficient was used to indicate the direction and strength of association between traits, with positive values indicating positive correlations and negative values indicating negative correlations.

Principal component analysis (PCA) was conducted to identify the major sources of phenotypic variation among the melon germplasm resources. Before PCA, all nine traits were standardized using Z-score transformation to eliminate the influence of different measurement units and scales. Principal components were extracted based on the eigenvalue greater than 1 criterion. The eigenvalue, contribution rate, cumulative contribution rate, and trait loadings of each principal component were calculated. PCA score plots and trait loading plots were generated to visualize the distribution of germplasm resources and the contribution of each trait to the principal components.

### 4.5. Cluster Analysis and One-Way ANOVA Test for Group Differences

Cluster analysis was performed based on nine quantitative DUS characteristics, including PL, IL, FLD, FTD, FPL, PT, MFT, SCD, and SSC. Before clustering, all traits were standardized using Z-score transformation to eliminate the influence of different units and scales. Euclidean distance was used to calculate the phenotypic distance among melon germplasm accessions, and hierarchical clustering was conducted using Ward’s minimum variance method, namely the Ward.D2 method. The clustering tree was cut into four groups to classify the 403 melon germplasm accessions into different phenotypic clusters.

To evaluate whether the classified groups differed significantly in quantitative DUS characteristics, one-way analysis of variance (one-way ANOVA) was performed for each trait using cluster group as the grouping factor. The significance level was determined based on the *p* value, and differences were considered significant at *p* < 0.05, highly significant at *p* < 0.01, and extremely significant at *p* < 0.001.

### 4.6. Construction of Comprehensive Selection Index

A comprehensive selection index was constructed to evaluate the overall agronomic performance of melon germplasm accessions based on nine quantitative DUS characteristics, including PL, IL, FLD, FTD, FPL, PT, MFT, SCD, and SSC. According to the breeding objectives, FLD, FTD, FPL, MFT, and SSC were defined as positive traits, whereas PL, IL, PT, and SCD were defined as negative traits. All traits were first standardized using Z-score transformation, and then adjusted according to their selection direction. Positive traits retained their standardized values, whereas negative traits were multiplied by −1.

Trait weights were assigned according to their relative importance in melon breeding. SSC was assigned the highest weight of 2.0; FLD, FTD, FPL, PT, MFT, and SCD were assigned weights of 1.0; and PL and IL were assigned weights of 0.5. The comprehensive selection index (SI) was calculated as follows:

$$SI=\sum_{i=1}^{n}Z_{i}\times W_{i}$$

where $Z_{i}$ represents the standardized value of the *i*th trait after directional adjustment, $W_{i}$ represents the corresponding trait weight, and n represents the number of traits. A higher Selection Index indicates better comprehensive agronomic performance and greater potential breeding value. All accessions were ranked according to the Selection Index to identify candidate germplasm resources with superior overall performance.

### 4.7. Data Analysis and Plotting

All raw phenotypic data were checked and organized before statistical analysis. For each accession and trait, individual plant or fruit measurements were averaged within each biological replicate. The two replicate-level values were subsequently averaged to obtain the annual mean, and the annual means from the three experimental years were further averaged to obtain the final three-year mean phenotypic value. These three-year mean values were used as the accession-level observations in all subsequent statistical analyses.

All data analyses and figure generation were performed using R software (v4.5.1). The raw phenotypic data were first checked and organized, and the mean value of each trait within biological replicates was used for subsequent analysis. The R packages tidyverse [47], ggplot2 [48], ggtree [49], ggtreeExtra [50], FactoMineR [51], factoextra [52], and treeio [53] were used for data cleaning, statistical analysis, visualization, correlation analysis, principal component analysis, and cluster analysis. All figures were exported in high-resolution formats for further editing and publication. Statistical significance was defined as *p* < 0.05, *p* < 0.01, and *p* < 0.001, corresponding to *, **, and ***, respectively. For visualization of trait distributions, histograms were normalized to probability density and overlaid with kernel density estimation curves. Therefore, the *y*-axis of Figure 2 represents probability density rather than the raw number or frequency of accessions within each interval.

## 5. Conclusions

In summary, this study provides a standardized quantitative phenotypic assessment of 403 melon accessions based on three-year DUS observations, revealing extensive variation and high diversity levels across quantitative traits. Multivariate statistical evaluations, including principal component and cluster analyses, successfully defined four distinct quantitative phenotypic groups under the tested environmental conditions, with fruit morphological structure, flesh thickness, rind thickness, seed cavity size, and soluble solids content serving as key drivers of germplasm differentiation. Furthermore, the application of a comprehensive selection index effectively prioritized high-ranking candidate accessions with strong breeding potential. Although this study does not establish the absolute genetic uniqueness of Xinjiang melon germplasm, it establishes an essential quantitative framework to support germplasm conservation, core collection construction, parental line selection, and new cultivar development. Future studies integrating multi-environment trials, qualitative descriptors, and molecular markers will be crucial to confirm the environmental stability, genetic basis, and practical value of these findings.

## Data Availability

The data provided in this study are available upon request from the corresponding authors. Due to privacy concerns, these data are not publicly available.

## Supplementary Materials

The following supporting information can be downloaded at: https://www.mdpi.com/article/10.3390/plants15152384/s1, Figure S1: Determination of the optimal number of clusters using average silhouette width. Average silhouette widths were calculated for cluster numbers ranging from k = 2 to k = 10 based on hierarchical clustering of 403 melon germplasm accessions using nine quantitative DUS traits. Points indicate the average silhouette width for each candidate number of clusters, and the connected line illustrates changes in clustering performance. Table S1 Phenotypic variation and descriptive statistics of nine quantitative DUS traits in 403 melon germplasm accessions. Table S2 Eigenvalues, individual contribution rates, and cumulative contribution rates of principal components based on nine quantitative DUS traits in 403 melon germplasm accessions. Table S3 Average silhouette width under different numbers of clusters. Table S4 Trait scores of nine quantitative DUS traits in 403 melon germplasm accessions.
